# Does China improve social welfare after implementing the national volume-based procurement?

**DOI:** 10.3389/fphar.2023.1178026

**Published:** 2023-10-11

**Authors:** Huan Wang, Ya-Tong Huo, Qian Zhuang

**Affiliations:** School of International Pharmaceutical Business, China Pharmaceutical University, Nanjing, China

**Keywords:** Chinese drug policy, national volume-based procurement, social welfare, consumer surplus, producer surplus, drug welfare

## Abstract

**Objective:** To explore the changes in social welfare before and after the implementation of the national volume-based procurement (NVBP). Explore whether the NVBP promotes the healthy development of manufacturers under the premise of benefiting patients. Then put forward relevant suggestions on how to effectively intervene the government in the pharmaceutical market.

**Methods:** Starting with consumer surplus and producer surplus, social welfare was studied from the three perspectives of price, supply, and demand.

**Results:** Consumer surplus was significantly increased, and the drug welfare of patients was significantly improved. The profits of the whole pharmaceutical industry have decreased but will increase in the future. The welfare of individual pharmaceutical enterprises varies. Overall social welfare has been significantly improved.

**Conclusion:** The core purpose of the NVBP is to improve the medication welfare of patients, and through the increase of consumer surplus, it can affect the increase of producer surplus. Under such a linkage mechanism, the diversified linkage system of “price, demand, and supply” will achieve the effect of “1 + 1+1 > 3”.

## 1 Introduction

From 2016 to 2020, China’s fiscal expenditure on health and wellness increased from 1315.9 billion yuan to 1754.5 billion yuan, with an average annual growth rate of 7.5% (Ministry of Finance of China, 2020). The cost of drugs is one of the most important components of health expenditure. Access to medicines, including availability and affordability, remains one of the biggest health challenges faced by all countries (Morgan et al., 2020). According to statistics, in 2020, the total cost of drugs accounted for 30.98% of the total national health expenditure that year, with *per capita* drug costs reaching 1,466.23 RMB (Li et al., 2022). Chinese medical institutions have long procured drugs through centralized bidding. However, the increasing autonomy of larger hospitals has resulted in a gradual interest chain among pharmaceutical enterprises driving up drug prices and decoupling between volume and price (where only prices increase without a corresponding increase in quantity), insufficient competition (as there is no obvious “patent cliff” phenomenon in China), and decentralized procurement at lower levels (Sun et al., 2023). To effectively address these issues, the General Office of the State Council of the People’s Republic of China issued the national volume-based procurement policy (NVBP) at the end of 2018.

As an essential measure to lower drug prices, ease the financial burden on patients, and benefit society as a whole, the NVBP has played an effective role in market regulation and government intervention (An et al., 2021; Zhao et al., 2021). The first round of the policy pilot was implemented in four municipalities and seven sub-provincial cities known as “4 + 7” policy: Beijing, Tianjin, Shanghai, Chongqing, Shenyang, Dalian, Xiamen, Guangzhou, Shenzhen Chengdu, and Xi’an (Zhu and Wang, 2023). It has undergone eight batches and nine rounds until 11 April 2023. By clarifying the specific volume of purchase and passing Generic Consistency Evaluation (GCE) as the finalist criteria, the price of winning the bid has been considerably reduced, and the policy of “volume for price” has been sincerely implemented (Luo et al., 2022).

Due to significant technical barriers, high risk, and market necessity, the pharmaceutical industry tends to exhibit features of monopolistic or monopolistic competition in economics (Lu et al., 2018). In order to expand their market share, pharmaceutical manufacturers heavily invest in drug sales and promotion activities, resulting in escalating drug prices (An et al., 2021). Consequently, monopolists generate excessive profits at the expense of patients who bear substantial drug costs, and his situation leads to market failure and a loss of social welfare known as deadweight loss (Huan et al., 2006). Therefore, government intervention is necessary to pursue the maximization of social welfare (Zhang, 2002). Welfare economics suggests that when engaging in market regulation, the government should consider both consumer surplus and producer surplus as they represent public interests with the aim of maximizing overall societal wellbeing (Gao, 2018).

Starting from the social utility and social cost, we explored the changes in social welfare before and after the NVBP policy. It examines whether these policies can promote productive enterprises while benefiting patients, and provides recommendations on effective intervention in the pharmaceutical market.

## 2 Materials and methods

### 2.1 Combine economic and policy perspectives

We explored the changes in social welfare before and after the NVBP policy in terms of two dimensions, social utility and social cost. In terms of social utility, the social welfare of the drug market was defined by referring to consumer surplus and producer surplus in microeconomics. Moreover, we analyzed the situation of drug market welfare before the NVBP policy, when the drug market tends to monopolize the market (Lu et al., 2018). After the NVBP policy, drug prices and drug costs dropped significantly. Firstly, the consumer surplus and producer surplus after the NVBP policy were analyzed in terms of drug price reduction and supply increase, and the cost reduction due to both changes. Secondly, on this basis, the social welfare changes brought about by the increase in drug demand (drug accessibility) were further analyzed. In terms of total social costs, static inefficiency and dynamic inefficiency were analyzed to highlight the change in social welfare.

The data used were from the Menet website, the Shanghai Sunshine Medical Purchase Network, and the Wind database. The Menet website is a leading comprehensive professional information service platform integrating medical and health industry, hospital market, retail market, business channel, internet online medical and health information services (https://www.menet.com.cn/). Recording the amount of bid-winning drug data and price data of the NVBP. The Shanghai Sunshine Medical Purchase Network is a website where the Chinese government publishes the NVBP policy and the bid-winning information, including the general name, specifications, supplier, and supply region of each batch of the NVBP (https://www.smpaa.cn/). The Wind database is a huge Chinese data platform involving global macro and industrial economies, including the Pharmaceutical Database, which contains data on drug sales from sample hospitals in China. The following data about drug demand were mainly from this database (https://www.wind.com.cn/).

### 2.2 Definition of social welfare in the drug market

Social welfare represents the utility level of social economic actors, divided primarily into consumers and producers. Cardinal utility, as the core of welfare assessment, is associated with the size and direction of national economic wellbeing (Ma and Shi, 2018). To effectively measure base utility, the concepts of consumer surplus and producer surplus have emerged. Currently, the sum of consumer surplus and producer surplus is commonly used to represent society’s net gain from transactions, with its changes roughly reflecting alterations in social welfare (Shi and Yang, 2020). Patients, as consumers, receive pharmaceutical benefits by alleviating or eliminating disease. They seek maximum health benefits at minimum economic cost to optimize subjective satisfaction (Xu and Tang, 2022). As producers, pharmaceutical manufacturers maximize their welfare when they achieve maximum profits and market share.

**FIGURE 1:**
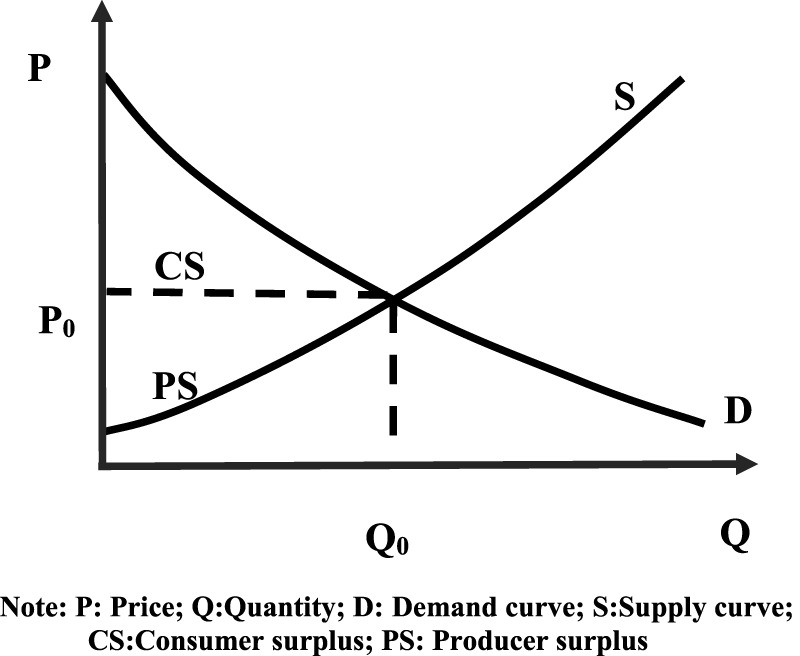
Consumer Surplus and Producer Surplus. Note: P: Price; Q:Quantity; D: Demand curve; MC:Marginal cost; MR:Marginal revenue; S represents the area of the graph, and A, B, C, D, etc., represent different areas.

Consumer surplus (***CS***) refers to the difference between what consumers are willing to pay for a product at most and what they really pay for it (Ni and Hu, 2014), while producer surplus (***PS***) is defined as the difference between what producers receive for a product and their lowest acceptable price supply (Gao, 2018). Equilibrium market prices are determined by market supply and demand dynamics. However, due to income disparities and individual preferences in the choice process among consumers, they may hold psychologically different price expectations. The difference between these two values constitutes the consumer surplus, which reflects the level of consumer satisfaction. As illustrated in Figure 1 (Gao, 2018), consumer surplus is expressed as the area below the demand curve ***D*** and above the market price ***P***_***0***_ (Tao, 2006). Similarly, the producer surplus is expressed as the area above the supply curve S and below the market price ***P***_***0***_.

To better evaluate the therapeutic value of medications for patients (represented as ***W***), the health benefits derived from medication are quantified in terms of their willingness to pay, which is reflected in the consumer price (represented as ***M***). It can also be stated that patients’ willingness to pay determines the consumer price. Patients incur economic costs to acquire drugs, namely, the product of drug price and quantity required (***P*** represents price and ***Q*** represents quantity). Additionally, patients bear time costs, transportation costs, and additional associated expenses (recorded as ***C***_***S***_) which affect their demand for drugs (Yin and Tang, 2019). Essentially, the current consumer price equals the initial consumer price minus time costs, transportation costs, and additional related expenses, which is:

$$M=M_{0}-C_{S}$$

The patient medication benefit is:

$$W=MQ-PQ=\left(M_{0}-C_{S}\right)Q-PQ=CS$$

The quantity a producer supplies is affected by the numerous costs of producing, transporting, advertising, selling, and so on (recorded as ***Cp***), and the lowest price a producer is willing to supply is when producer profit (recorded as) is zero, and the market price is equal to the average cost of production. Therefore, the profit of the producer is:

$$\pi =\left(P-C_{P}\right)Q=PS$$

## 3 Results

### 3.1 Social welfare analysis before national volume-based procurement

In 2015, the National Medical Products Administration (NMPA) issued the Opinions on Promoting Drug Price Reform. The policy abolished the government pricing system that had been in place for nearly 2 decades, resulting in a gradual shift of the dominant influence over drug prices back to the market. During the same year, the National Health and Family Planning Commission released the Guiding Opinions of the General Office of State Council on Enhancing Centralized Procurement of Drugs in Public Hospitals, which concentrated the government’s supervision of drug prices in the procurement link.

In theory, the manufacturer, as the recipient of the market price, can maximize social welfare in a perfectly competitive market (Tang et al., 2015). However, in a monopolistic market where manufacturers face a downward-sloping demand curve instead of a fixed supply curve, profit maximization necessitates equality between marginal revenue (***MR***) and marginal cost (***MC***), namely, ***MR = MC*** (Shen and Yu, 2005). Marginal Revenue refers to the increased revenue generated by each additional unit of product sales, while Marginal Cost refers to the increase in cost generated by each additional unit of output (Gao, 2018). When ***MC*** equals ***MR***, the marginal profit becomes zero. That is to say, the incremental profit brought by each incremental unit of output of a firm is zero, indicating that the firm has reached the maximum profit at this time (Shen and Yu, 2005). For some time now, public medical institutions with greater information resources and pharmaceutical manufacturers with higher technical barriers have dominated both medical service provision and drug supply markets. As a result, they exert control over drug pricing, leading to monopolistic or oligopolistic economic behavior that leads to inefficiencies such as high drug prices, low supply, and excessive monopoly profits resulting in deadweight loss. There is room for Pareto improvement in the market, and the efficiency and fairness of the drug market need to be improved urgently (Gao, 2018).

From the patients, due to information asymmetry, much of the autonomy and choice to use drugs rests with doctors and pharmaceutical manufacturers. Therefore, drug prices include numerous additional costs besides production costs and transportation costs, such as search costs, negotiation costs, sales costs, transaction costs, etc. In addition, pharmaceutical manufacturers link the performance of doctors with the use of drugs to occupy a large market share in medical institutions, which gives rise to “gray space” that creates inflated drug prices (Jiang et al., 2021). However, the essence of drug price formation is the market mechanism. This process reflects the market failure of monopoly behavior which results in deadweight loss of social welfare.

**FIGURE 2:**
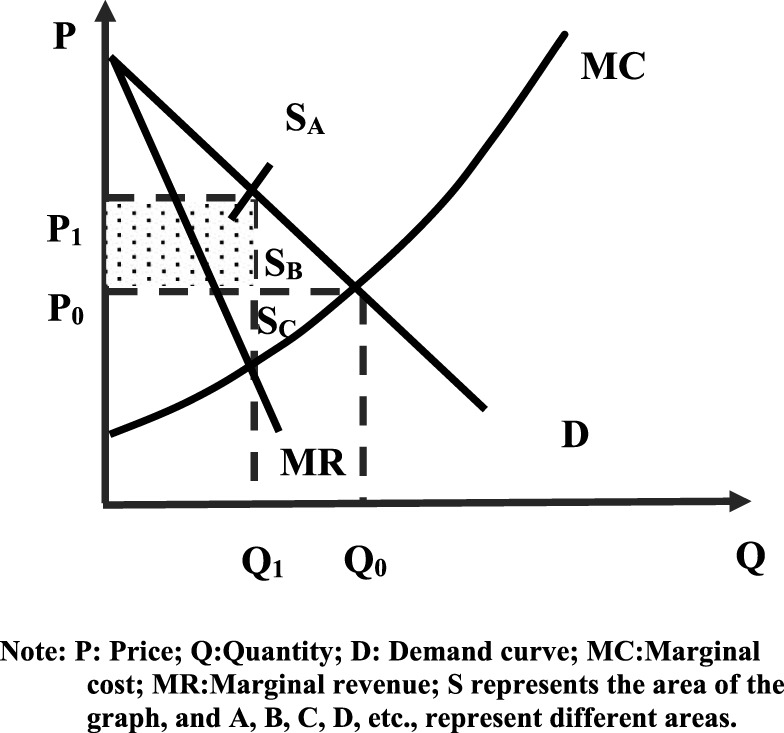
Social welfare before volume-based purchase. Note: P: Price; Q: Quantity; D: Demand curve; MC:Marginal cost; MR:Marginal revenue; S represents the area of the graph, and A, B, C, D, etc., represent different areas.

Figure 2 shows the deadweight loss of social welfare. Compared with the price ***P***_***0***_ and output ***Q***_***0***_ in the perfectly competitive market equilibrium, the monopolistic competitive market has a higher price ***P***_***1***_ in and lower production ***Q***_***1***_. The consumer surplus has also been reduced by an increase in the price of drugs, which has reduced the area $S_{A}$ and area $S_{B}$ (***S*** represents the area of the graph, and ***A, B, C, D***, etc., represent different areas), then the producer surplus has changed:

$$PS=S_{A}-S_{C}$$

The final change in social welfare (recorded as ***W***_***F***_) is:

$$W_{F}=\left(-S_{A}-S_{B}\right)+\left(S_{A}-S_{C}\right)={-S}_{B}-S_{C}$$

Therefore, the social welfare loses $S_{B}+S_{C}$ due to monopolistic behavior, which results in a deadweight loss.

The reduction in consumer surplus $S_{A}$ is due to high drug prices, where patients pay more for the same number of drugs. However, this increases the burden of drugs on the patient and affects the patient’s consumption concept. The reduced consumer surplus $S_{B}$ is due to high prices and patients giving up the quantity purchased. The burden of drugs for patients is further increased, and some patients cannot afford drugs, which directly endanger their life safety. The increase in the producer surplus $S_{B}$ is a complete shift away from the consumer surplus. The increase in high monopoly profits may aggravate the phenomenon that the pharmaceutical industry only pursues profit while ignoring the quality of drugs and life safety, which is not conducive to the healthy development of the pharmaceutical industry. The reduced producer surplus $S_{C}$ and the consumer surplus $S_{B}$ become the welfare loss of the whole society. That is why China should promote Pharmaceutical Volume-Based Procurement.

### 3.2 Factors affecting social welfare from national volume-based procurement

The model of drug purchasing with quantity aims to solve the “separation of bidding and purchase” and “separation of quantity and price” under the centralized purchasing system (Zhu et al., 2019). The separation of drug bidding and purchase gives medical institutions the chance of “second price negotiation”. With the quantity of drugs purchased by medical institutions unclear, there is poor information between hospitals and drug manufacturers, and prices and supplies are subsequently affected. Pharmaceuticals are purchased in quantity to reasonably control drug costs, reduce the burden of medication on patients, and reduce the cost of market operations for enterprises. In addition, the policy can guide hospitals and doctors to use drugs rationally (Li and Bai, 2019; Chen et al., 2021; Yuan et al., 2021). From the consumer surplus and producer surplus, the impact of bulk purchasing on social welfare is mainly focused on drug cost price and drug accessibility.

#### 3.2.1 Drug prices and costs

The NVBP has achieved certain results since its implementation in 2018, with the drug varieties continuously expanding and the number of winning enterprises increasing. Some drug prices were cut by as much as 98 percent. A total of 60 drugs won the bidding in the seventh batch of drug procurement, covering 31 therapeutic categories and the average price reduction for selected drugs was 48 percent (Peng and Qiu, 2022). On average, five firms were selected for each drug variety. Drug prices tend to be reasonable, and supply diversification and stability are further enhanced. Behind the highly compressed price of drugs is a dramatic reduction in the cost to drugmakers. The bid-winning enterprises obtain a large share of orders through centralized one-time purchases, directly saving the transaction and sales costs, including search costs, negotiation costs, contracting costs, supervision costs, etc. (Xu, 2011). In addition, pharmaceutical enterprises no longer need to “bribe” hospitals which squeeze the gray space in the process of drug circulation, largely reduces the market operation costs of pharmaceutical enterprises and effectively reduces drug prices and costs.

Take the first batch of “4 + 7” procurement of bid-winning drugs as an example, 25 pilot drugs in the “4 + 7” expansion of procurement were all successful. Moreover, their price compared with the “4 + 7” pilot selected price level, an average reduction of 25% (Zhang, 2019). The bid-winning price and the price decrease for some drugs are shown in Table 1, with relevant data from Menet and Shanghai Sunshine Pharmaceutical Purchasing website. As can be seen from the table, most drugs have been reduced in price to varying degrees, with Rosuvastatin Calcium Tablets being reduced by 74.31%. In addition, after the announcement of the “4 + 7” centralized procurement results, some regions required the enterprises that failed to win the bid to implement a gradient price reduction before they could continue to purchase online, which also affected the price of the original drugs that failed to win the bid. The gradient price reduction results of some unbid-winning original drugs are shown in Table 1 (Wang et al., 2021). It can be seen that the price of each original drug has decreased to varying degrees, which indicates that the implementation of the volume-based purchase policy not only reduces the price of the bid-winning enterprise but also has a certain impact on the price of other unsuccessful enterprises, effectively controlling the price of the whole pharmaceutical market.

**TABLE 1:** Price for some bid-winning drugs of “4 + 7” centralized procurement and unbid-winning original drugs (box/RMB).

Generic name of drug	Specifications	Price of “4 + 7” collection	Price of “4 + 7” expanded	Reduction (%)	Original drug price before “4 + 7”	Price after “4 + 7”	Reduction (%)
Atorvastatin Calcium Tablets	20 mg*7 tablets	6.60	3.84	41.82	56.00	42.77	23.63
Rosuvastatin Calcium Tablets	10 mg*28 tablets	21.80	5.60	74.31	196.00	155.12	20.86
Clopidogrel Bisulfate Tablets	75 mg*7 tablets	22.26	17.81	19.99	107.10	91.70	14.38
Escitalopram Oxalate Tablets	10 mg*7 tablets	30.94	27.86	9.95	95.20	81.13	14.78
Olanzapine Tablets	10 mg*7 tablets	67.51	43.60	35.42	142.80	123.48	13.53
Gefitinib Tablets	250 mg*10 tablets	547.00	257.00	53.02	-	-	-
Sodium Fosinopril Tablets	10 mg*14 tablets	11.80	11.80	0.00	-	-	-
Lisinopril Tablets	10 mg*28 tablets	6.45	6.45	0.00	-	-	-
Enalapril Maleate Tablets	10 mg*16 tablets	8.93	8.93	0.00	19.20	18.24	5.00
Levetiracetam Tablets	250 mg*30 tablets	72.00	71.79	0.29	138.00	117.90	14.57
Montelukast Sodium Tablets	10 mg*5 tablets	19.38	18.96	2.17	38.00	28.80	24.21
Montmorillonite Powder	3 g*15 bags	10.20	4.16	59.22	27.00	21.60	20.00
Pemetrexed Disodium for Injection	100 mg/count	810.00	798.00	1.48	3030.00	2676.58	12.53
Pemetrexed Disodium for Injection	500 mg/count	2776.97	2735.83	1.48	10450.00	9176.27	12.19
Flurbiprofen Axetil Injection	5 mL:50 mg* 5pcs	109.75	109.40	0.32	-	-	-
Dexmedetomi-dine Hydrochloride Injection	2 mL:0.2 mg* 4pcs	532.00	532.00	0.00	-	-	-

According to the categories of drugs winning the bidding and their proportion in the total drug sales of the enterprise, Huahai Pharmaceutical and Jingxin Pharmaceutical became the greatest winners in the “4 + 7” collection and “4 + 7” expansion (Wang et al., 2021). Huahai Pharmaceutical has six and seven bid-winning varieties in these two volume purchases, respectively, with the bid-winning variety accounting for 87.59 percent of the 2019 volume. Jingxin Pharmaceutical also had three winning bids, with its 2019 bidable varieties accounting for 49.93 percent of the total sales. Therefore, Huahai Pharmaceutical and Jingxin Pharmaceutical can be selected as representative enterprises to analyze the cost changes. Related data comes from Menet’s pharmaceutical enterprise data and financial data from the company’s annual report. See Table 2 for details.

**TABLE 2:** Some financial data of the two enterprises before and after “4 + 7” centralized procurement (million RMB).

Enterprises	Expense items	2020	Year-on-year growth (%)	2019	Year-on-year growth (%)	2018	Year-on-year growth (%)
Huahai Pharmaceut-ical	Operating income	6485.21	20.36	5388.09	5.76	5094.6	1.85
	Selling expenses	996.33	3.73	960.54	25.79	1294.37	43.08
	Advertising expenses	786.61	14.48	687.11	30.96	995.21	35.95
	Research and development expenses	565.7	21.04	467.36	17.79	396.76	-
Jingxin Pharmaceutical	Operating revenue	3258.08	10.66	3646.68	23.88	2943.8	32.66
	Selling expenses	965.1	28.03	1341.06	21.85	1100.57	61.92
	Advertising expenses	3.54	6.95	3.31	25.62	4.45	78.93
	Research and development expenses	259.24	2.02	254.10	4.99	242.02	47.30

Table 2 shows that after purchasing volume, the growth rate of sales expenses for both companies decreased significantly, with Huahai Pharmaceutical experiencing negative growth in 2019 compared to 2018 by 25.79 percent. Advertising and promotion expenses for the two companies in 2019 showed negative growth of 25–30 percent compared to 2018 and were highly influenced by the volume of purchases. The reduction in their sales expenses was reflected in lower drug costs.

**FIGURE 3:**
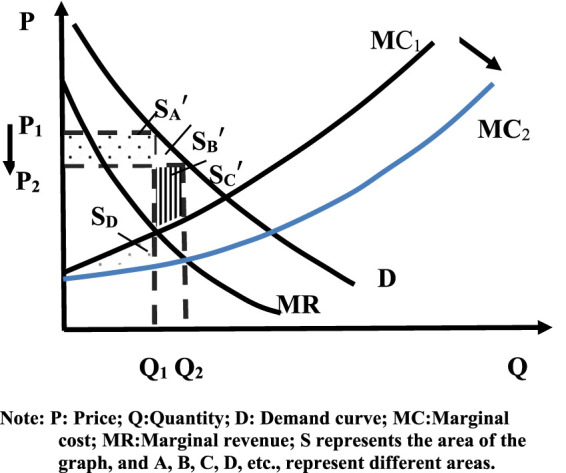
Social welfare of changes in drug price and cost after volume-based purchase.

From an economic point of view, falling prices will cause businesses to reduce their supply (Gao, 2018). However, as reflected in the centralized procurement of the pharmaceutical market, price declines will lead to increased supply. Because drugs are necessities for patients, the price elasticity of demand is narrow, that is, the rate of increase of demand is less than the rate of decline of price, which is reflected in the supply side that the rate of decline of supply is less than the rate of decline of price (Zhu and Gu, 2006). In addition, the NVBP policy directly stipulates that the winning enterprises can obtain a large market share of hospitals at low prices and increase the supply volume of the enterprises (Ma et al., 2022). This, in turn, will help the winning company generate economies of scale and reduce variable costs beyond fixed costs such as consistency assessment, resulting in a reduction in the winning company’s marginal costs. However, in order to win the remaining drug market share, the winning companies will voluntarily lower their prices and pursue economies of scale to maintain certain revenues. As a result, the marginal cost of the entire drug market is on a downward trend. The marginal cost (***MC***) curve moves downward and becomes slower (see Figure 3), as can be seen from Figure 3, after the implementation of volume-based procurement, drug prices decreased from ***P***_***1***_ to ***P***_***2***_ and the production of drug market increased from ***Q***_***1***_ to ***Q***_***2***_ due to the effect of the market mechanism. Drug prices are in the ***P***_***1***_ ***-P***_***2***_ range if drug company costs stay the same. Due to the decrease in drug prices after the NVBP policy, the cost of drugs has been squeezed, forming a chain reaction of drug price reduction and drug cost reduction. Thus, the shift in social welfare is explored by combining drug prices and marginal cost reductions. Reduced drug prices allowed consumer surpluses to grow:

$$CS=S_{A}^{\prime }+S_{B}^{\prime }$$

And the producer surplus changes:

$$PS=S_{C}^{\prime }+S_{D}-S_{A}^{\prime }$$

Eventually social welfare increases:

$$W_{F}=S_{B}^{\prime }+S_{C}^{\prime }+S_{D}$$

The deadweight loss caused by the monopoly compared to before the volume-based purchase was changed from the original ***S***_***B***_ ***+ S***_***C***_ to ***S***_***B***_ ***+ S***_***C***_ ***-S***_***B***_ ***' -S***_***C***_ ***'***. It shows that volume-based purchasing can increase social welfare by reducing the deadweight loss caused by monopoly. In addition, the producer surplus (the area of ***S***_***D***_) can be added due to the reduction in marginal cost. The monopoly profit (the area of ***S***_***A***_ ***'***) of the producer is transformed into the drug benefit of the patients. Patients can achieve greater health benefits while reducing the financial burden of medication and increasing their trust in drugs, medical institutions, and even the state.

#### 3.2.2 Drug accessibility

Drug accessibility refers to the extent to which a drug is safely and effectively available to patients at a reasonable price, including quantity, space, and time (Yin and Tang, 2019; Jiang and Tan, 2022). Factors affecting drug accessibility include not only drug price but also drug supply and the convenience of obtaining drugs (Yin and Tang, 2019). Therefore, the accessibility of drugs explored in this paper mainly focuses on the price, quality, quantity, and convenience of drugs available in a medical facility over a certain period of time. According to volume-based procurement, a pharmaceutical company supplies a certain drug to a medical institution with a specific market share, emphasizing the production capacity and supply guarantees of the bidding company. Then government departments systematically manage the drug circulation link. Pharmaceutical enterprises enhance the stability and accessibility of drug supply based on cost reduction. Consistency assessment of generic drugs is a threshold for pharmaceutical companies to participate in volume-based purchasing. On the one hand, it stimulates enterprise and increases the availability of such drugs. On the other hand, generic drug prices can fall off a cliff while maintaining quality, effectively improving access to medical care. Procurement by volume realizes the distribution of different enterprises in different regional markets and coordinates the allocation of medical resources to the maximum extent. It ensures that the same or similar bid-winning drugs are available in different regions and improves the ease of access. All these factors effectively improve the accessibility of purchasing drugs in quantity. In addition, the synergistic effect of purchasing with quantity and the medical insurance payment system also improves the accessibility of drugs from the perspective of remote reimbursement and the expansion of the reimbursement ratio (Tan et al., 2021).

**FIGURE 4:**
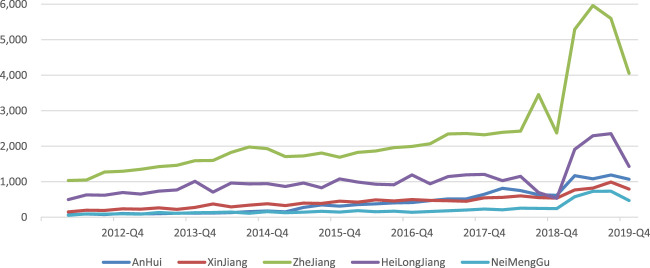
Sales of Atorvastatin Calcium Tablets in sample hospitals of some provinces (ten thousand RMB). Note: P: Price; Q: Quantity; D: Demand curve; MC: Marginal cost; MR: Marginal revenue; S represents the area of the graph, and A, B, C, D, etc., represent different areas.

**FIGURE 5:**
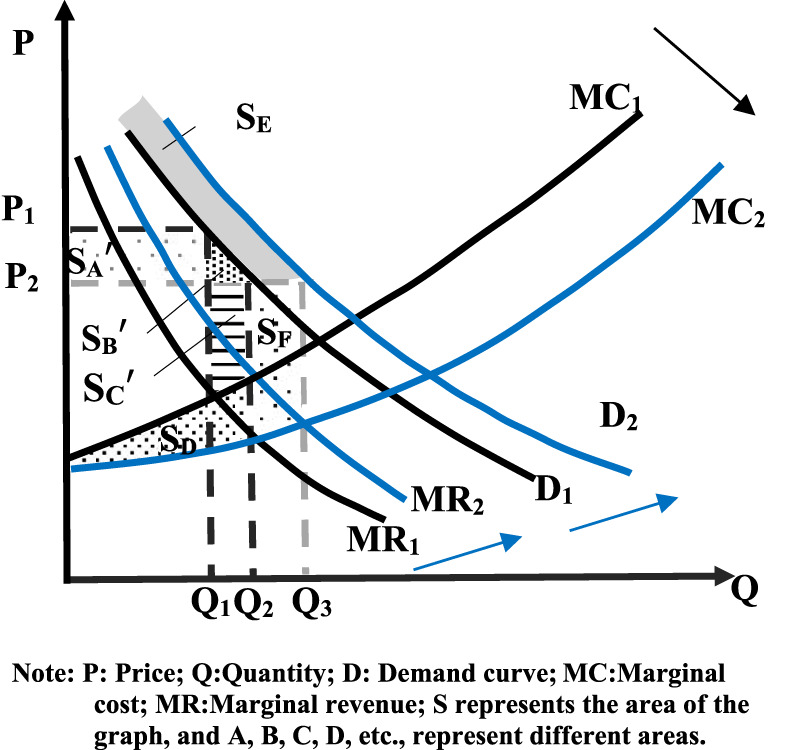
Social welfare of changes in drug accessibility after volume-based purchase (Case 1: P unchanged). Note: P: Price; Q: Quantity; D: Demand curve; MC: Marginal cost; MR:Marginal revenue; S represents the area of the graph, and A, B, C, D, etc., represent different areas.

The increased availability of drugs affects patient purchasing behavior. When patients can quickly and conveniently access and afford better quality drugs, patients’ willingness to pay will increase accordingly, thus increasing patient demand for drugs. Take the winning bid of Atorvastatin Calcium Tablets purchased in “4 + 7” pilot centralized volume-based procurement as an example (relevant data from the Wind database). Compared with before the “4 + 7” pilot centralized volume-based procurement, the sales volume of sample hospitals in some provinces increased significantly, indicating that the use of this drug in hospitals increased significantly. Figure 4 shows the result. Patient demand for drugs increases, and thus the demand curve in Figure 5 is shifted to the upper right.

In a monopoly market, the effect of changes in the demand curve on prices and quantities is uncertain. Reducing the price of drugs purchased in quantity is a prerequisite, and changes in demand are a chain effect. Therefore, this paper deals with the effect of changing demand on social welfare in two cases. Case 1 is when the price of the drug decreases and the marginal cost decreases while demand increases but the price remains the same. This is because the government has increased regulation to control spontaneous regulation of the market. In Case 2, demand increases and prices fall more after drug price decrease and marginal cost decrease. Only the market adjusts spontaneously.

Figure 5 shows that based on the price decrease and marginal cost reduction, the price remains unchanged at *P* *_2_* and the drug market output increases from ***Q***_***2***_ to *Q*_*3*_ after the demand increases. At this point, the consumer surplus increases as compared to before the purchase of the quantity:

$$CS=S_{A}^{\prime }+S_{B}^{\prime }+S_{E}$$

Producer surplus changes:

$$PS=S_{C}^{\prime }+S_{D}+S_{F}-S_{A}^{\prime }$$

And eventually social welfare increases:

$$W_{F}=S_{B}^{\prime }+S_{C}^{\prime }+S_{D}+S_{E}+S_{F}$$

In addition, if only demand changes (when the demand curve ***D1→D2***, the supply is ***MC2,*** without regard to ***MC1→MC2***), consumer surplus and producer surplus increase by ***S***_***E***_ and ***S***_***F***_, respectively. If only supply changes (when marginal cost ***MC1→MC2***, the demand is ***D2***, do not consider the demand curve ***D1→D2***), this can only make the producer surplus change ***S***_***F***_. It can be found that consumer surplus can drive producer surplus to a certain extent, which means that governments focus on increasing consumer surplus when formulating and improving social welfare. Consumer demand can drive supply and thus increase enterprise profits.

**FIGURE 6:**
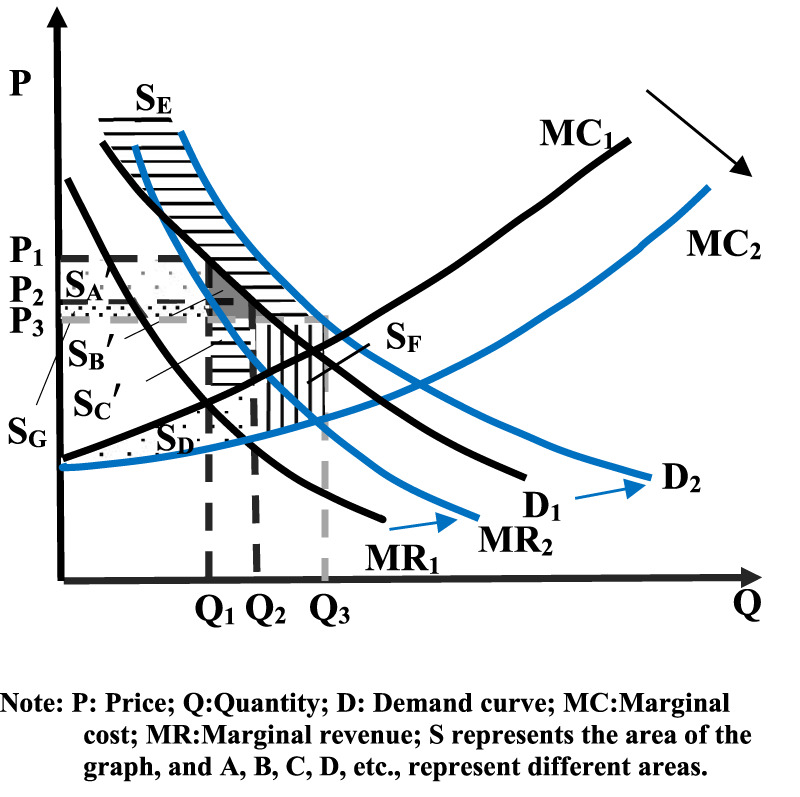
Social welfare of changes in drug accessibility after volume-based purchase (Case 2: P decreased). Note: P: Price; Q: Quantity; D: Demand curve; MC: Marginal cost; MR: Marginal revenue; S represents the area of the graph, and A, B, C, D, etc., represent different areas.

Figure 6 shows that after the increase in demand, price further decrease from ***P***_***2***_ to ***P***_***3***_, and drug production increases from ***Q***_***2***_ to ***Q***_***3***_ due to spontaneous market regulation. At which point the consumer surplus increases:

$$CS=S_{A}^{\prime }+S_{B}^{\prime }+S_{E}+S_{G}$$

Producer surplus change:

$$PS=S_{C}^{\prime }+S_{D}+S_{F}-S_{A}^{\prime }-S_{G}$$

Social welfare finally increases:

$$W_{F}=S_{B}^{\prime }+S_{C}^{\prime }+S_{D}+S_{E}+S_{F}\left(\text{similar to case }1\right)$$

However, the further decline in prices means that the producer surplus of the *S* *_G_* portion is converted into the consumer surplus, which does not affect the final change in social welfare.

From ***S***_***C***_ ***'+S***_***D***_ ***- S***_***A***_ ***'*** in Figure 3 to ***S***_***C***_ ***'+ S***_***D***_ ***+ S***_***F***_ ***-S***_***A***_ ***'*** in Figure 5 to the ***S***_***C***_ ***'+ S***_***D***_ ***+ S***_***F***_ ***- S***_***A***_ ***' -S***_***G***_ in Figure 6, it can be found that there is a reduction in both parts, and the reduced part is converted into consumer surplus. However, whether the final producer surplus increases or decreases under different circumstances remains to be discussed. The average price reduction for the seven batches of drugs was 53 percent, with the maximum price reduction exceeding 95 percent. As an example, the sales volume of the bid-winning drug in the second quarter of 2019 and the fourth quarter of 2018 are shown. Sales rose for five drugs, such as Irbesartan Tablets and Levetiracetam Tablets. The other 21 drugs had a downward trend after the implementation of the “4 + 7” centralized procurement, with an average decrease of 47.54% (Wang et al., 2021). Table 2 shows the year-over-year decline in the operating income of Jingxin Pharmaceutical. Sanofi’s Clopidogrel Bisulfate Tablets were not included in the first round of “4 + 7” purchases, and sales in 11 pilot cities from April to October 2019 fell 14% compared with the same period last year. This indicates that volume-based purchasing has considerably compressed the overall profit of the pharmaceutical industry. While it has also compressed various production costs, the range of cost reductions is limited. As a result, the revenue reduction from the effect of lower prices far outweighs the cost reduction. It indicates that the surplus of producers in ***S***_***A***_ ***'*** that changes due to falling prices is considerably larger than the surplus in ***S***_***C***_ ***' +S***_***D***_ ***+ S***_***F***_ that changes due to lower costs or increased demand. The producer surplus is reduced, and the welfare of the pharmaceutical market as a whole is reduced. However, the increase or decrease in benefits for individual businesses is uncertain. The pharmaceutical market is divided into bid-winning and non-bid-winning companies. For non-selected enterprises, the lack of market share of medical institutions has considerably squeezed their living space. Faced with a limited surplus market share and low prices, its profits have fallen and the producer surplus is bound to shrink. However, the continuous implementation of volume-based procurement encourages non-selected enterprises to carry out consistent evaluation and improve drug quality, effectively standardizing competition in the drug market. Moreover, this policy motivates quality optimization under generic conformity assessment and the transformation of generic drugs into innovative drugs. It can improve production efficiency and achieve a reasonable allocation of resources in the pharmaceutical market. The difference between ***S***_***A***_ ***'*** and ***S***_***C***_ ***' +S***_***D***_ ***+ S***_***F***_ of producer surplus becomes smaller. Finally, an increase in the final producer surplus is achieved. Social benefits will be increased further.

### 3.3 Analysis of total social welfare

**FIGURE 7:**
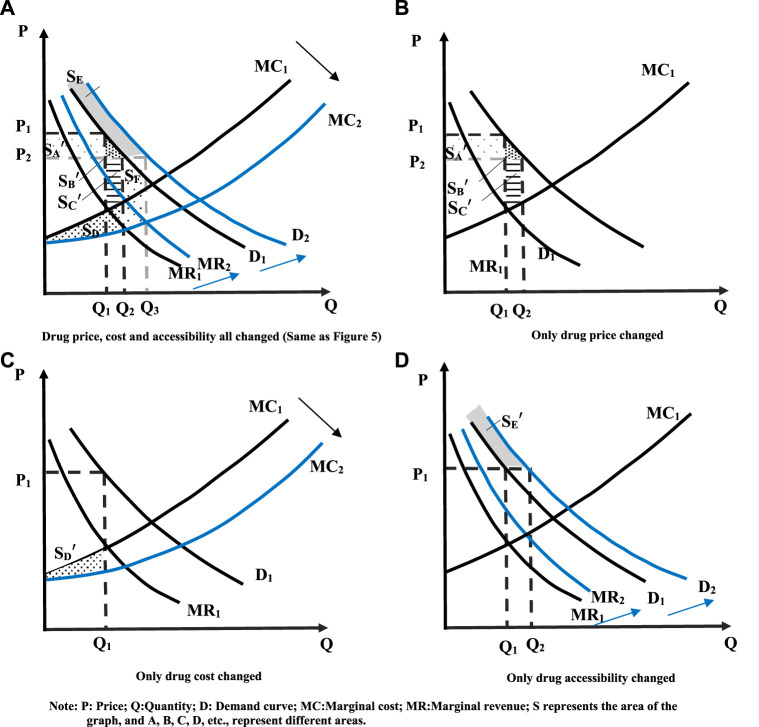
“1 + 1+1 > 3” effect. **(A)** Drug price, cost and accessibility all changed (Same as Figure 5); **(B)** Only drug price changed; **(C)** Only drug cost changed; **(D)** Only drug accessibility changed.

The increase in social welfare can be discussed in terms of total social utility and total social costs. The third part of the paper focuses on the variation of the total social utility. Firstly, it analyzed the changes in consumer and producer surpluses resulting from drug price and cost reductions, and highlights the changes in total social utility brought about by patients and pharmaceutical companies. Based on this, the change in social welfare from drug availability was further discussed. Combined with the above results, it can be seen that the decrease in price and cost and the increase in demand can increase social welfare. The increase in social welfare due to the combination of the three effects is greater than the sum of the increases due to each of the three effects. Figure 7 shows the variation of the specific social welfare. A simultaneous change in price, cost, and demand increases the total utility of social welfare by $S_{B}^{\prime }+S_{C}^{\prime }+S_{D}+S_{E}+S_{F}$ (as shown in Figure 5), while only a decline in price increases social welfare by $S_{B}^{\prime }+S_{C}^{\prime }$ . When only the cost decreased, social welfare increased $S_{D}^{\prime }$ ***,*** and $S_{D}^{\prime }<S_{D}$ . When only demand increases, social welfare increases $S_{E}^{\prime }$ , and $S_{E}^{\prime }<S_{E}$ . Therefore $S_{B}^{\prime }+S_{C}^{\prime }+S_{D}+S_{E}+S_{F}>S_{B}^{\prime }+S_{C}^{\prime }+S_{D}^{\prime }+S_{E}^{\prime }$ **,** it can be said that the superposition of the three effects produces 1 + 1+1 > 3.

Alternatively, the change in total social welfare can be discussed in terms of social costs. Total social cost decreases and social welfare increases, while total social cost consists of static inefficiency cost and dynamic inefficiency cost (Liu et al., 2013). Static efficiency refers to neglecting the costs required by technological changes and innovations of pharmaceutical companies and focusing only on the optimal allocation of resources at the current stage. In the pharmaceutical market after the NVBP policy, it has been shown that the excess profits of pharmaceutical companies are reasonably compressed and the burden of drugs on patients is reduced. Dynamic efficiency aims to focus on the ability of enterprises to create resources and coordinate resources, which is demonstrated by the enthusiasm and possibilities of pharmaceutical companies for R&D innovation in the pharmaceutical market after the NVBP policy, and the initiative and innovation of coordinating resources (Tang and Xu, 2008).

The NVBP policy improves the static efficiency of the current drug market and reduces the static inefficiency cost. On the one hand, government agency hospitals use the bidding mechanism to conduct volume procurement, increase the level of reasonable competition among enterprises in the pharmaceutical market, and improve the efficiency of resource allocation in the existing pharmaceutical market. On the other hand, the market-led, government-assisted drug pricing and trading mechanisms have begun to mature through generic drug consistency assessment as a condition for purchasing quantity, ensuring the quality of drugs and establishing a fair price competition platform for drug evaluation (Wang et al., 2022).

The NVBP policy has improved the dynamic efficiency of the long-term development of the pharmaceutical market and reduced the cost of dynamic inefficiency. For generic drugs that have not been evaluated, enterprises have taken a major market share in order to enter volume purchasing, which will further stimulate enterprises to research and develop generic drugs, and constantly innovate the process of manufacturing and efficacy of generic drugs. For companies that have evaluated generic drugs but failed to win bids, they can pursue the remaining market share by innovating sales channels and coordinating production resources on the one hand. On the other hand, it can focus on the development of new drugs and capture the market for innovative medicines.

**FIGURE 8:**
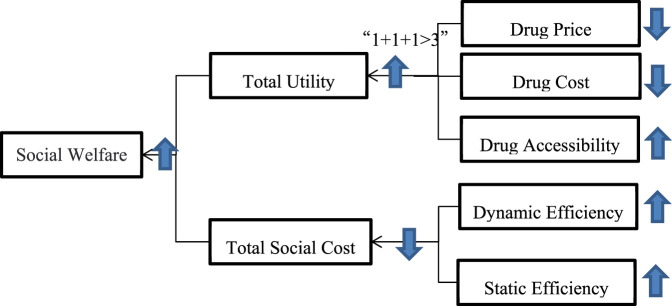
Social welfare change logic.

In summary, after the NVBP policy, both static and dynamic efficiency increase, the total social cost decreases, and the social welfare increases. Therefore, the improvement in social welfare is reflected in an increase in the total utility of the drug market and a reduction in the total cost. The specific logic of the change in social welfare is shown in Figure 8.

The implementation of the NVBP has an effect on patients and pharmaceutical enterprises. The burden of drugs on patients was reduced. From the existing case data, the *per capita* drug cost of the winning drug has decreased significantly, the health effect has increased, and the drug benefit of patients has been greatly improved (Shen et al., 2021). The overall surplus of the pharmaceutical industry shows an increasing trend, which ultimately promotes the balanced allocation of medical resources.

At the present stage, non-selected enterprises are facing the dilemma of losing market share and declining profit. Selected companies face marginal profit declines after economies of scale. The effective way is to take the innovative path. How can the government ensure the rational use of drugs at low prices while encouraging innovation in research and development by enterprises? Moreover, pharmaceutical enterprises how to optimize the development direction is an important problem to be solved in the future.

## 4 Conclusion and recommendations

In this paper, we explore the change in social welfare before and after procurement with quantity through consumer surplus and producer surplus, and show that the implementation of procurement with quantity can promote Pareto improvement and increase social welfare. At the same time, however, it is facing problems such as the survival plight of non-selective businesses with limited numbers and low prices. In view of this, together with the results of this paper, the following proposal is made.

### 4.1 Take the medication welfare of patients as the starting point to innovate the multi-party linkage mechanism

The original intention of the volume procurement model is to achieve a substantial reduction in drug prices and bulk supply through government intervention in drug prices, so as to reduce the burden of drug use for patients and promote rational drug use. The present study finds that the increase in consumer surplus can contribute to the increase in producer surplus to a certain extent and play a linkage role. In the future, when formulating relevant policies, the government should first consider the welfare of patients to enable people to use higher-quality drugs at lower prices, obtain higher health effects, and improve the overall level of medication. In this way, the government should first focus on price and quality. When it comes to the community interests of all parties, the government should stick to the bottom line of low prices and high quantities, improve collection rules, and support policies and overall working mechanisms. More importantly, the government should be guided by clinical needs to maximize the benefits of medicines for patients through quality and efficacy consistency assessments.

Second, the welfare of other stakeholders should be taken into account when increasing patient benefits. For pharmaceutical companies, the increase in patient welfare will drive an increase in demand, and they can increase their producer surplus driven by patient welfare. In the short term, profits are slight and sales are high. The winning company obtains the vast majority of the market share of the hospital and the majority of the monopoly profits. In the long run, the promotion of the overall level of socialized medicine will drive the redistribution of domestic medical resources to promote corporate innovation and research. And, it will speed up the transformation and upgrading of the pharmaceutical industry so that enterprises can gain greater core interests. For hospitals, the implementation of purchasing with quantity reduces the original “grey space”. It seems to be detrimental to the increase of the welfare of hospitals and medical staff, but the saved medical insurance funds can be used for more treatment services and innovative medical devices. The key resources can be invested in more urgent medical needs (Li and Bai, 2019). The government should also introduce relevant policies to benefit pharmaceutical enterprises and hospitals under volume-based procurement, maintain a reasonable competitive market environment, and promote the development of hospitals.

### 4.2 “Price, demand, and supply” should be combined to achieve the effect of “1 + 1+1 > 3”

In the volume-based purchase mode, the effect of drug price reduction, demand increase, and supply increase (cost reduction) is superimposed, resulting in a greater social welfare increase than the social welfare increase under the three actions, respectively, showing the effect of “1 + 1+1 > 3”. In the future, when the government improves the volume-based procurement mode, it should increase social welfare virtually from the three of “price, demand, and supply.”

The decrease in drug prices and costs is mainly due to the elimination of sales and transaction costs, and the compression of the grey space in the profit chain of hospitals and pharmaceutical companies. Integrated supervision should be implemented from drug production to distribution. And, enterprises should strengthen supply compliance to eliminate additional costs beyond the cost of manufacturing and transportation of medicines. At the same time, the welfare of patients and the profits of pharmaceutical companies should be guaranteed.

Pharmaceutical companies should establish supply mechanisms to ensure the timely and adequate supply of drugs. For those unable to complete the procurement schedule, the government should increase penalties, guide rational competition in the pharmaceutical industry, and strengthen the spirit of enterprise contracts (Negera et al., 2021). Include supply indicators as one of the bidding requirements in the evaluation system for hospitals and pharmaceutical companies.

In addition to reducing the burden of drugs on patients, volume-based procurement increases the availability of bid-winning drugs, which is conducive to increased demand for drugs. Governments should increase the availability and demand for drugs by considering the range of diseases to be treated, clinical needs, applicability, and universality. As much as possible, the centralized procurement of medicines should be coordinated with the list of essential medicines, the list of medicines covered by medical insurance, and the list of medicines in short supply, and more collective purchases of medicines should be added.

### 4.3 Focus on the surplus market and non-selected enterprises and encourage enterprise innovation

Most of the increase in producer surplus after the NVBP comes from the monopoly profits of the selected firms, while for a large number of non-selected firms, volume-based purchasing leaves them facing a survival dilemma caused by the loss of hospital market share. Non-chosen companies have to turn to the surplus market. Under the double pressure of rigid price reduction and quality improvement constraints, non-selective enterprises are at risk of market elimination. Therefore, the government should retain some surplus market share and guarantee living space for some non-selected enterprises based on flexible policy requirements. And encourage them to produce superior technological generic drugs and innovative drugs with broad prospects to meet the diversified needs of the current pharmaceutical market.

The state actively introduces relevant policies to encourage pharmaceutical innovation by considering the long-term development of pharmaceutical enterprises and the diversified needs of patients in the future under the NVBP policy. Non-selected enterprises can prioritize the development of generic drugs and strive for market share in the NVBP after passing the consistency evaluation. The government can provide support in terms of project establishment and allocation of special financial funds to expand the choice space in the centralized procurement of drugs, which can promote healthy competition and further benefit patients. Non-selected enterprises can also change their strategic goals, and like selected enterprises, they can actively develop innovative drugs, lay out other disease areas, and take the lead in seizing market share. Government departments can provide technical support, financial special fund allocation, tax incentives, and other financial and technical encouragement policies for innovative drugs, and policies such as drug priority review and approval can be introduced to help enterprises carry out the listing of innovative drugs.

## 5 Discussion

This study explored the changes in social welfare before and after the NVBP policy from the perspective of economics, focusing on factors such as drug price, cost, and accessibility. We systematically deduced how the NVBP policy improves social welfare from social utility and total social cost.

Our main innovation is that medicines, especially those under the policy of mass procurement, are different from other products as a special commodity. Under market regulation, most commodities will experience a decrease in production due to a decrease in price. However, due to the regulation of volume procurement policies, the drug production of enterprises will actually increase, thereby changing the derivation of social welfare results. We aim to emphasize this point and make it graphically concrete. At the same time, it is also our innovation to discuss the changes in social welfare before and after the NVBP policy from social utility, including consumer and producer surplus, and social cost. Furthermore, we put forward the “1 + 1+1 > 3” effect. The increase in social welfare due to the combination of the three effects is greater than the sum of the increases due to each of the three effects, which has guiding and practical significance for future policymaking.

However, our study is biased towards theoretical derivations. There are limitations in the use and methodology of the data, and there is a lack of data for the whole process of drug production to consumption to demonstrate changes in social welfare. In the future, based on the theoretical derivation of this study, additional studies can be researched. Data can be collected for the whole process from the hospital to the consumption of drugs, and consumer surplus and producer surplus can be expressed in terms of specific data. Time series analysis can be chosen to explain how the impact of NVBP on social welfare evolves over time.

## Data Availability

Publicly available datasets were analyzed in this study. This data can be found here: Menet Website; Wind Database; Shanghai Sunshine Medical purchase network.
